# Corded and Hyalinized Endometrioid Endometrial Carcinoma: A Rare Case Treated With Robot-Assisted Surgery

**DOI:** 10.7759/cureus.62274

**Published:** 2024-06-12

**Authors:** Hiroo Kawashima, Takeshi Fukuda, Kaori Sakamoto, Makoto Yamauchi, Toshiyuki Sumi

**Affiliations:** 1 Department of Obstetrics and Gynecology, Graduate School of Medicine, Osaka Metropolitan University, Osaka, JPN; 2 Department of Diagnostic Pathology, Graduate School of Medicine, Osaka Metropolitan University, Osaka, JPN

**Keywords:** carcinosarcoma, robot-assisted surgery, minimally invasive surgery, corded and hyalinized endometrioid carcinoma, endometrial carcinoma

## Abstract

Endometrial carcinoma is the sixth most common cancer among women worldwide. Minimally invasive surgery (MIS) has become the preferred treatment, offering similar survival outcomes to laparotomy with lower complication rates. Corded and hyalinized endometrioid carcinoma (CHEC) is a rare and diagnostically challenging variant of endometrioid carcinoma, first described in 2005, characterized by a biphasic appearance of traditional low-grade endometrioid adenocarcinoma and corded and spindled cells embedded in a hyaline stroma. A 55-year-old nulligravid woman presented with abnormal genital bleeding for 10 days. Initial evaluations, including transvaginal ultrasonography and histological examination, confirmed adenocarcinoma. Imaging studies (magnetic resonance imaging [MRI] and computed tomography [CT]) revealed a thickened endometrium (11 mm) with no myometrial invasion, enlarged pelvic lymph nodes, or distant metastasis. Tumor markers were within normal ranges. She underwent robot-assisted laparoscopic total hysterectomy, bilateral adnexectomy, and pelvic lymph node biopsy using the da Vinci Xi system (Intuitive Surgical, Sunnyvale, CA). Histopathological examination revealed CHEC, with characteristic epithelioid and spindled cells arranged in cords within a hyalinized stroma. Immunohistochemical staining showed focal positivity for cytokeratin AE1/AE3, weak estrogen receptor positivity, and nuclear β-catenin expression, distinguishing it from carcinosarcoma. The diagnosis was confirmed as CHEC, FIGO 2008 stage IA (pT1aN0M0). The patient remained disease-free 18 months post-surgery. CHEC is a rare variant of endometrioid carcinoma with unique histological features. It typically presents in younger patients at an early stage and has a favorable prognosis. Accurate diagnosis is crucial to differentiate it from more aggressive tumors like carcinosarcoma, preventing overtreatment. The immunohistochemical profile, particularly nuclear β-catenin accumulation, is useful in distinguishing CHEC from carcinosarcoma. This is the first documented case of CHEC successfully treated with robot-assisted surgery. Increased awareness among pathologists and clinicians is essential for accurate diagnosis and optimal management of this rare tumor variant.

## Introduction

Endometrial carcinoma is the sixth most common cancer in incidence among women worldwide, according to 2022 cancer statistics [1]. Minimally invasive surgery (MIS) has produced equivalent survival outcomes to laparotomy, with lower rates of intra- and postoperative complications leading to faster recovery and shorter hospital stays [2]. Specifically, robotic-assisted MIS offers mechanical assistance and support for surgical instruments, creating a more ergonomic and less fatiguing experience for surgeons. Additionally, the learning curve for robotic surgery is shorter [3,4]. Consequently, the rate of endometrial cancer treated with MIS has gradually increased, approaching 90% at high-volume centers [5].

The most predominant histological type of endometrial carcinoma is endometrioid carcinoma, accounting for up to 80% of cases [6], followed by serous carcinoma (10%) [7], clear-cell carcinoma (<10%) [8], and mucinous carcinoma, which is very rare [9] and is not well documented in terms of prevalence. While most endometrioid carcinomas are straightforward to diagnose, some histological variants pose diagnostic challenges. One such rare and diagnostically challenging variant is corded and hyalinized endometrioid carcinoma (CHEC). First described by Murray et al. in 2005, CHEC is a variant of endometrioid carcinoma characterized by a biphasic appearance, containing both traditional low-grade endometrioid adenocarcinoma and corded and spindled cells embedded in a hyaline stroma [10]. CHEC is a rare condition with an exact incidence rate that remains undetermined due to its relatively recent identification. Clinically, CHEC typically occurs in younger patients compared to typical endometrioid carcinoma, with an average age of approximately 49 years [11]. It has been reported that 68% of CHEC cases were found at the International Federation of Gynecology and Obstetrics (FIGO) stage I, and 84% were confined to the uterus, leading to a favorable prognosis with 70% of patients alive with no evidence of recurrence at the last follow-up [12].

We report a rare case of CHEC successfully treated with robot-assisted surgery. This is the first documented case of CHEC treated with this method.

## Case presentation

**Figure 1:**
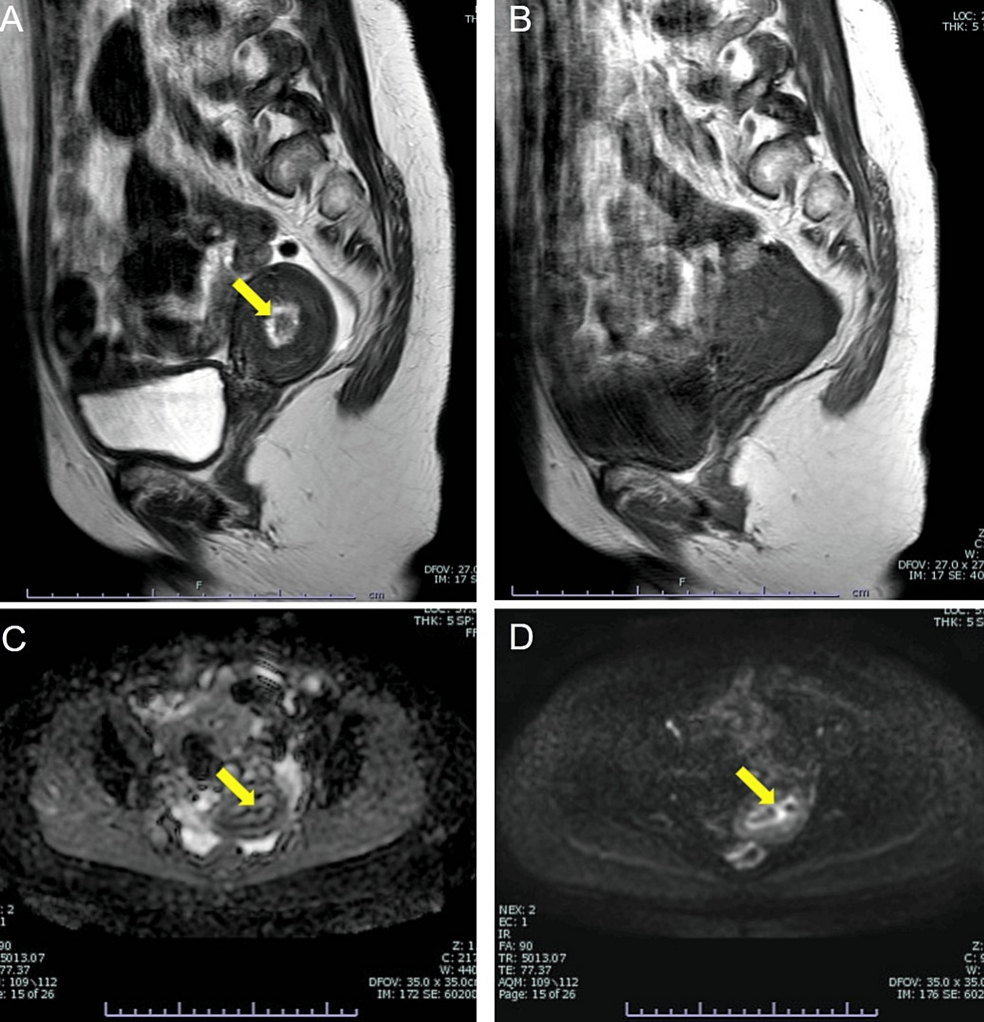
Magnetic resonance imaging (MRI) scans of a uterine tumor. T2-weighted image (T2WI) showing an 11-mm-sized tumor in the uterine cavity with isointensity. (A) The yellow arrow indicates the tumor. T1-weighted image of the same slice as in T2WI, showing the tumor. (B) Diffusion-weighted image (DWI) showing high intensity in the tumor, indicated by the yellow arrow (C). (D) Apparent diffusion coefficient (ADC) map showing low intensity in the tumor, indicated by the yellow arrow.

A 55-year-old nulligravid woman presented with abnormal genital bleeding for 10 days and consulted her gynecologist. The patient's medical history included Cornelia syndrome, schizophrenia, hypertension, and chronic heart failure. Due to Cornelia syndrome, she had mild mental retardation. Transvaginal ultrasonography revealed endometrial thickening; endometrial histology confirmed adenocarcinoma, prompting her referral to our hospital. A vaginal speculum examination showed a small amount of brownish vaginal discharge. Transvaginal ultrasonography indicated a thickened endometrium measuring 15 mm, with no bilateral ovarian enlargement and a small amount of ascites. Cervical cytology showed no intraepithelial lesion or malignancy (NILM). Magnetic resonance imaging (MRI) revealed an 11-mm-thickened endometrium with an intact junctional zone, indicating no myometrial invasion (Figure 1).

**Figure 2:**
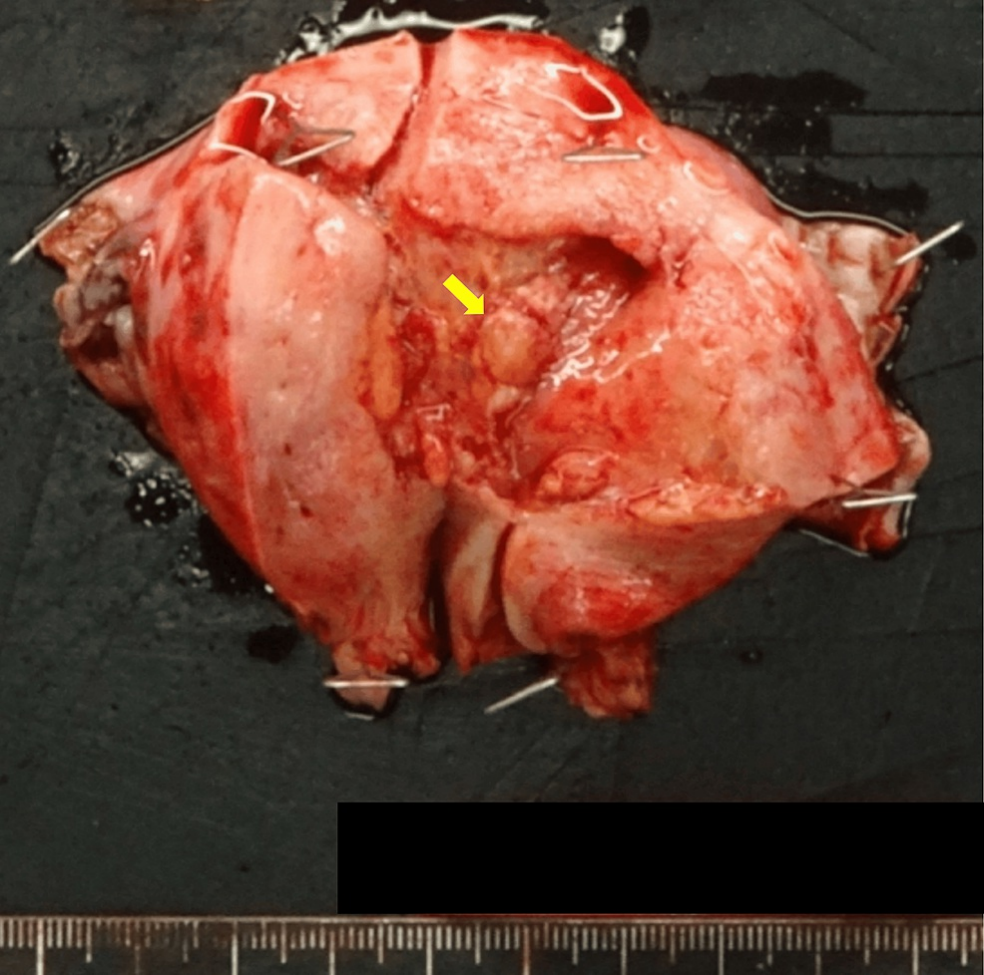
A gross image of a resected uterus. Polyp-like masses up to 10 mm × 8 mm × 4 mm were noted in the posterior wall of the uterine corpus. The yellow arrow indicates the tumors.

There were no enlarged pelvic lymph nodes or ascites. A computed tomography (CT) scan showed no distant metastasis. Tumor markers were within normal ranges: carcinoembryonic antigen (CEA) at 3.2 ng/mL (normal ≤ 5.0 ng/mL), CA19-9 at <2 U/mL (normal ≤ 37.0 U/mL), and CA125 at 14.16 U/mL (normal < 35.0 U/mL). Her medical history includes Cornelia syndrome, schizophrenia, hypertension, and chronic heart failure. She was stable in managing her schizophrenia, understood her condition, and provided written informed consent for treatment and publication of this case report. The patient underwent robot-assisted laparoscopic total hysterectomy, bilateral adnexectomy, and pelvic lymph node biopsy using the da Vinci Xi system (Intuitive Surgical, Sunnyvale, CA). The surgery was performed with four ports for the robot and one port for the assistant, arranged horizontally at the level of the umbilicus. The camera port was positioned in the umbilicus. No uterine manipulator was used during the surgery. The hysterectomy specimen revealed yellow polyp-like masses up to 10 mm × 8 mm × 4 mm in the posterior wall of the uterine corpus (Figure 2).

**Figure 3:**
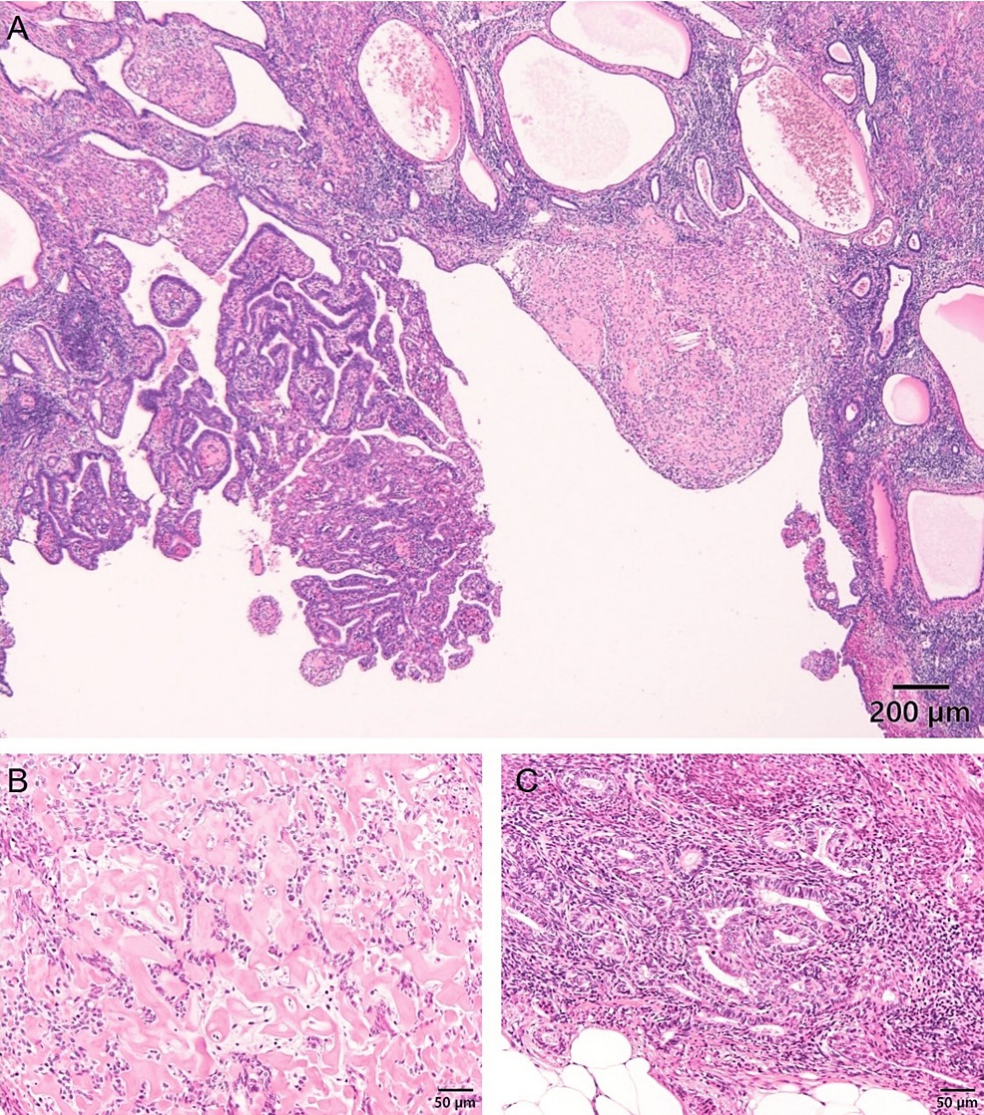
Hematoxylin-eosin stains of the tumor. Endometrioid glands and focally hyalinized area. (A) Scale bar: 200 µm. Epithelioid cells are arranged in cords within a hyalinized stroma. (B) Scale bar: 50 µm. Typical endometrioid carcinoma (Grade 1). (C) Scale bar: 50 µm.

Histologically, low-power views showed circumscribed nodules with hyalinization in the endometrium (Figure 3).

**Figure 4:**
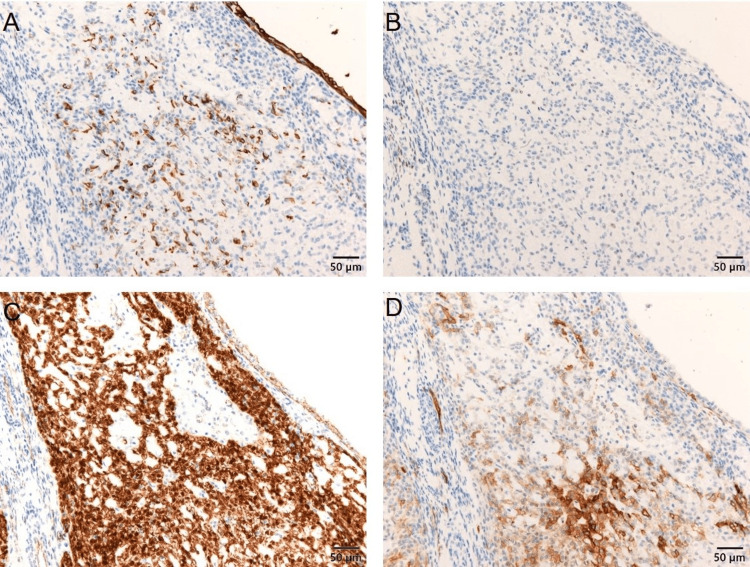
Immunohistochemical findings of the tumor. (A) Cytokeratin (AE1+AE3) is focally positive. (B) Estrogen receptor (ER) shows slight positivity in some cells. (C) β-Catenin is expressed in the nuclei of corded cells in the sex cord-like area. (D) Focal membranous expression of CD10. Scale bar: 50 µm.

High-power views revealed small epithelioid cells arranged in cords within a hyalinized collagenous stroma, with scant cytoplasm and round nuclei with mild atypia. No mitotic figures were observed. Neoplastic endometrioid glands, some adjacent to the hyalinized nodules, were also present. Immunohistochemical staining showed focal positivity for cytokeratin AE1/AE3 in epithelioid cells, weak focal positivity for estrogen receptor (ER), and nuclear expression of β-catenin in corded cells (Figure 4).

Inhibin, p53, and desmin were negative. Although CD10 showed partial membranous expression, endometrial stromal sarcoma was ruled out due to nuclear positivity of β-catenin. No metastasis was found in the resected lymph nodes. The diagnosis was CHEC based on histological and immunohistochemical findings, specifically FIGO 2008 [13] stage IA (pT1aN0M0). The patient remained alive without recurrence 18 months post-surgery.

## Discussion

Surgery is the most effective strategy for treating endometrial carcinoma, with the extent of surgery determined by the histological type and FIGO stage, as assessed through endometrial biopsy and imaging examinations such as MRI or CT [14]. For stage I and low-risk histological types, such as grade 1 or 2 endometrioid carcinoma, the standard procedure is total hysterectomy and bilateral salpingo-oophorectomy, with or without lymphadenectomy [15]. MIS has become the preferred method for this disease, supported by evidence from randomized controlled trials that demonstrate similar overall survival and recurrence rates, along with lower postoperative morbidity [14,15]. Robotic surgery, in particular, is conducted at lower pneumoperitoneum pressure, thanks to mechanical assistance from robotic arms [16]. As a result, robotic-assisted MIS has the potential to extend the benefits of the minimally invasive approach to a broader range of patients, including those at high risk of anesthetic complications such as obesity, the elderly, and individuals with medical comorbidities [4]. In the current case, the patient has comorbidities including Cornelia syndrome, which causes mental retardation, schizophrenia, hypertension, and chronic heart failure. Given these conditions, a MIS is the preferred procedure for treatment.

CHEC is a rare and distinctive variant of endometrioid carcinoma, characterized by unique histological features. This carcinoma exhibits cords, nests, or clusters of epithelioid and spindled cells embedded within a hyalinized stroma. These cellular components merge imperceptibly with a conventional low-grade endometrioid component, creating a biphasic appearance that can sometimes complicate diagnosis [10]. Histologically, CHEC is notable for its low-grade features, with epithelioid and spindled cells typically showing minimal nuclear atypia and a low mitotic index. The corded and hyalinized components are often superficially located and non-myoinvasive, suggesting that these tumors do not exhibit the aggressive behavior seen in other high-grade endometrial carcinomas. The low-grade characteristics of the endometrioid component help differentiate CHEC from more aggressive forms, such as carcinosarcoma, which typically show high-grade epithelial and mesenchymal components with significant nuclear atypia and mitotic activity [10]. The immunohistochemical profile of CHEC frequently includes nuclear β-catenin accumulation. This marker is useful in distinguishing CHEC from carcinosarcoma, as the latter usually does not exhibit this nuclear staining pattern [12,17]. Molecular studies, particularly those guided by the Cancer Genome Atlas classification, reveal that most CHEC cases fall into the *no specific molecular profile* subgroup, further aligning it with low-grade endometrioid carcinomas [11,18]. Clinically, CHEC tends to present in younger patients compared to typical endometrioid carcinoma, with a mean age of approximately 49 years [11]. Most CHEC cases are diagnosed at an early stage (FIGO stage I or II), and the tumors are generally confined to the uterus. The prognosis for CHEC is generally favorable, with a high percentage of patients remaining disease-free at follow-up [12]. Despite its favorable prognosis, CHEC can pose significant diagnostic challenges. Its biphasic morphology, characterized by both epithelioid and mesenchymal elements, can lead to misdiagnosis as carcinosarcoma, particularly in biopsy or curettage specimens. Carcinosarcomas are typically high-grade and clinically aggressive, so misdiagnosis can lead to overtreatment [10,11]. Awareness and accurate recognition of CHEC are, therefore, crucial for appropriate patient management.

## Conclusions

This is the first case report of CHEC successfully treated with robot-assisted surgery. CHEC is an uncommon variant of endometrioid carcinoma, characterized by distinctive histological and molecular features. It primarily affects younger patients, often presenting at an early stage with a generally favorable prognosis. Accurate diagnosis, facilitated by recognizing its unique histological patterns and immunohistochemical markers, is essential to differentiate it from more aggressive tumors such as carcinosarcoma and to guide appropriate clinical management. Increased awareness among pathologists and clinicians is crucial to avoid misdiagnosis and ensure optimal treatment for patients with this rare tumor variant. Additionally, due to its favorable prognosis, MIS is suitable for this condition if detected at an early stage.
